# Urothelial bladder afferents selectively project to L6/S1 levels and are more peptidergic than those projecting to the T13/L1 levels in female rats

**DOI:** 10.1016/j.heliyon.2023.e18495

**Published:** 2023-07-20

**Authors:** Buffie Clodfelder-Miller, Jennifer J. DeBerry, Timothy J. Ness

**Affiliations:** a Department of Anesthesiology and Perioperative Medicine, University of Alabama at Birmingham, Birmingham, AL, UK

**Keywords:** Bladder, Primary afferents, CGRP, Substance P

## Abstract

This neuroanatomical study in four, adult, Sprague-Dawley female rats quantified the number of Urothelial (labeled by intravesical DiI dye administration) and Non-Urothelial (labeled by intraparenchymal injection of Fast blue dye) bladder primary afferent neurons (bPANs) located in the T13, L1, L6 and S1 dorsal root ganglia. Additional immunohistochemical labeling using antibodies to detect either Substance P or CGRP further characterized the bPAN samples as peptidergic or non-peptidergic. Cell counts indicated that Urothelial bPANs were more common at the L6/S1 levels and more likely to be identified as peptidergic when compared with bPANs characterized at T13/L1 levels and with Non-Urothelial bPANs. These studies provide additional evidence that at least two distinct neuronal populations, with differing localization of sensory terminals, differing peptide content, and differing projections to the central nervous system, are responsible for bladder sensation.

## Highlights

• Bladder primary afferent neurons (bPANs) at T13/L1 and L6/S1 levels were compared.
• Injection: intravesical DiI (Urothelial); intraparenchymal Fast Blue (Non-Urothelial).
• Urothelial bPANs more common at L6/S1 levels than T13/L1 levels Urothelial bPANS more likely to be immunopositive for SP/CGRP at L6/S1 levels Non-Urothelial bPANs were similar at L6/S1 and T13/L1 levels.

## 1 Introduction

Recent studies have identified that lumbosacral bladder primary afferents (bPANs) in rats [1] and mice [2] with bare endings in the suburothelial space are phenotypically different from bPANs with endings located in the rest of the bladder. Termed Urothelial and Non-Urothelial bPANs respectively, these populations of neurons have been shown to be anatomically distinct since the intravesical administration of a neuronal tracer dye labels Urothelial bPANs and intraparenchymal injections of a neuronal tracer dye labels Non-Urothelial bPANs with little overlap. Patch-clamp studies of the two different neuronal populations in mice have demonstrated them to have different electrophysiological properties [2] and calcium imaging studies in rats have demonstrated lesser responsiveness of Urothelial bPANs to TRPA1 agonists [1] when compared with Non-Urothelial bPANs. The Urothelial bPANs have particular value to translational research because they can be reached and biopsied cystoscopically in humans (e.g., as in Ref. [3]) without significant morbidity, whereas biopsy of muscular or serosal layers is generally not performed due to risk of bladder perforation. Hence, to understand the properties of urothelial afferents is of high clinical importance.

Previous basic science studies have often limited study of bPANs to those with cell bodies residing in the L6 and S1 dorsal root ganglia (DRG) despite known bladder primary afferents with cell bodies in the lower thoracic and upper lumbar dorsal root ganglia [4]. Based on histological axon-tracing studies, it is not known if Urothelial bPANs are unique to lumbosacral (LS) levels since most studies of bPANs have utilized intraparenchymal injections of retrograde labeling agents (e.g. Ref. [5]) and those techniques were not likely to have labeled the Urothelial bPANS. In our previous studies in rats and mice [1,2], we demonstrated that the retrograde dyes Fast Blue and DiI, when injected intraparenchymally, do not label most, if any, of the Urothelial bPANs when compared against neurons backlabeled by intravesically administered agents. We have also demonstrated that the intravesically administered dyes do not spread beyond the submucosal levels [1,2].

Neuropeptides such as Calcitonin Gene-Related Peptide (CGRP) and Substance P (SP) are known to be present in afferents innervating the bladder. However, it is not known whether CGRP and/or SP have any selective expression in Urothelial versus Non-Urothelial bladder afferents. Therefore, the present study sought to simultaneously determine whether expression of CGRP and SP differed between these two neuronal populations in any spinal level-dependent fashion. This was done by performing experiments in which DRG neurons were simultaneously retrogradely labeled from intravesical and intraparenchymal dye injection sites in the bladder and then also examined, using immunohistochemical methods, for either CGRP or SP content.

## 2 Materials and methods

### 2.1 Animals

Subjects were four female Sprague Dawley rats (200–250 g; Invigo, Sprattville, AL). All procedures were approved by the UAB IACUC (protocol 22026). bPANs were retrogradely labeled using Fast Blue (FB; Polysciences Inc.; 1% w/v solution in sterile water) injected into the bladder wall and by 1,1′-dioctadecyl-3,3,3′,3′-tetramethylindocarbocyanine perchlorate (DiI; Molecular Probes; 1 mg/ml solution in 10% DMSO in sterile saline) infused into the lumen of the bladder as previously described [1]. FB labeling was always performed on the day prior to DiI infusion and tissues were extracted one week later under deep anesthesia and following exsanguination. T13, L1, L6 & S1 DRG were dissected out bilaterally, fixed in 4% paraformaldehyde overnight and cryoprotected in 30% sucrose. Serial interrupted sections (cryostat - 10 μm) were slide mounted, rinsed in 0.1 M PB, permeabilized with 0.1% Triton X-100, rinsed again, and incubated in blocking buffer containing 2% bovine serum albumin. Tissues were then incubated overnight at 4°C in blocking buffer containing the following primary antibodies: polyclonal rabbit anti-CGRP primary antibody (1:2000; ImmunoStar cat. #24112) or polyclonal guinea pig anti-SP primary antibody (1:1000; Abcam cat. #10353). The following day, tissues were rinsed and incubated with either AffiniPure donkey anti-rabbit FITC-conjugated secondary antibody (1:200; Jackson ImmunoResearch cat. #711-095-152) or AffiniPure donkey anti-guinea pig FITC-conjugated secondary antibody (1:200; Jackson ImmunoResearch cat. #706-095-148). Images for analyses were obtained using a Nikon Ti80E microscope and a 10× dry objective. Only cells with a nucleus in the plane of the photomicrograph were analyzed and areas calculated for each cell in μm^2^. Area was converted to diameter for presentation and analysis. ImageJ software was utilized for analysis and strict criteria for labeling were employed with background fluorescence measured at five random sites and averaged to serve as a criterion level for sampling: all neurons labeled as “positive” had fluorescence intensities >5 SD above the mean of the background sites. All data are represented as mean ± SEM unless noted. One- and two-way ANOVAs followed by Tukey's HSD were used to compare the number of neurons and their diameters. Fisher's exact tests were used to compare distributions of labeling characteristics in different neuronal populations.

## 3 Results

### 3.1 General

**Fig. 1:**
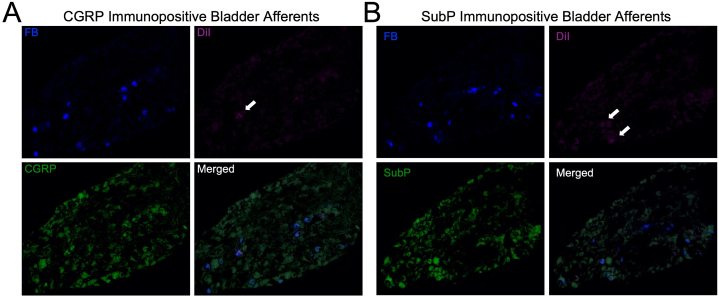
Representative examples of dorsal root ganglion neurons retrogradely labeled with Fast Blue and DiI, and immunohistochemically stained with antibodies against CGRP (Panel A) and SubP (Panel B). White arrows indicate DiI-labeled neurons. (For interpretation of the references to colour in this figure legend, the reader is referred to the Web version of this article.)

**Fig. 2:**
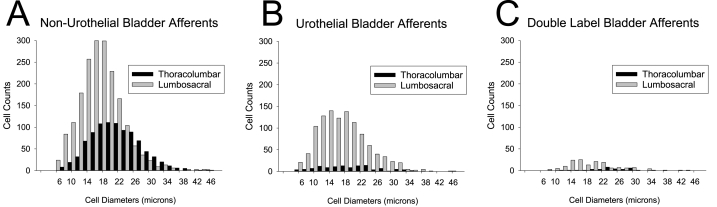
Total cell counts for thoracolumbar (T13/L1) and lumbosacral (L6/S1) dorsal root ganglion neurons stratified according to size. Panel A indicates neurons labeled by intraparenchymal injections of Fast Blue into the urinary bladder wall. Panel B indicates neurons labeled by the intravesical instillation of DiI. Panel C indicates neurons labeled by both methods. See text for more complete description.

**Fig. 3:**
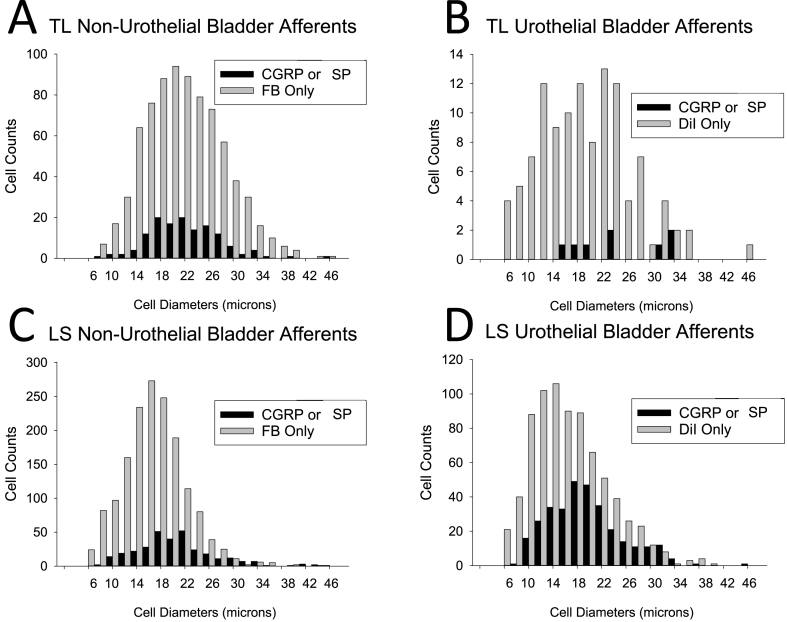
Total cell counts for thoracolumbar (T13/L1) and lumbosacral (L6/S1) dorsal root ganglion neurons stratified according to size and peptide content. Panels A & C indicate neurons labeled by intraparenchymal injections of Fast Blue into the urinary bladder wall. Panels B & D indicate neurons labeled by the intravesical instillation of DiI. Note differences in Y-axis ranges. Data from neurons labeled by both methods are not presented due to their small number. See text for more complete description.

The sample of DRG neurons used for analyses was limited to those demonstrating retrograde labeling from the bladder. The total number of bPANs characterized in this study was 4215, with 1069 neurons identified in the T13/L1 (thoracolumbar) ganglia and 3146 neurons identified in the L6/S1 (lumbosacral) ganglia. A summary of the cell counts and their mean sizes are given in Table 1. Representative examples of neuropeptide immunopositive, retrogradely labeled neurons are shown in Fig. 1. A graphical comparison of different labeled neuronal subgroups stratified according to spinal levels and cell size is given in Fig. 2. As is apparent, the cell size data are normally distributed. As is apparent from the data in Table 1 – there was very little difference in the size of neurons identified as having CGRP content and those identified as having SP content, although the SP-containing neurons were slightly larger in most groups. Due to their similarity, the data from these two separate samples were merged and are described as “peptidergic” neurons for purposes of graphical presentation in Fig. 3 and statistical analysis.

**Table 1:** Cell Count; Cell Diameters (μm±SEM) of bPANs.

Group Designation	Non-Urothelial Only		Urothelial Only		Double Labeled	
Labelling →	FB Only	FB + Peptide	DiI Only	DiI + Peptide	DiI + FB	DiI + FB + Peptide
DRG Level-IHC eval						
T13-SP stain	160; 24.5 ± 0.5	22; 28.3 ± 1.3	30; 24.8 ± 1.0^@^	6; 27.2 ± 2.7	3; 27.4 ± 2.2	8; 25.6 ± 1.2
T13-CGRP stain	140; 23.4 ± 0.6	36; 25.7 ± 1.5	23; 18.3 ± 1.2	0	0	3; 29.7 ± 1.8
L1-SP stain	283; 24.7 ± 0.4^@@^	44; 23.4 ± 0.8	19; 23.4 ± 1.6^@@^	2; 26.9 ± 6.2	2; 28.9 ± 1.8	9: 28.3 ± 1.8
L1-CGRP stain	197; 21.7 ± 0.4	33; 21.4 ± 0.8	41; 17.7 ± 1.1	0	3; 26.2 ± 2.9	5; 21.8 ± 2.0
Total TL	780; 23.7 ± 0.3**	135; 24.4 ± 0.6*	113; 21.3 ± 0.9**	8; 27.1 ± 2.4*	8; 27.3 ± 1.3**	25; 26.4 ± 1.0*
L6-SP stain	392; 17.8 ± 0.2	57; 20.5 ± 0.6	275; 19.0 ± 0.4^@^	119; 20.5 ± 0.5	33; 18.3 ± 0.6	11; 22.1 ± 1.5
L6-CGRP stain	445; 17.6 ± 0.2	85; 20.2 ± 0.6	279; 17.6 ± 0.4	111; 20.9 ± 0.7	25; 17.3 ± 0.8	35; 21.6 ± 1.5
S1-SP stain	363; 19.8 ± 0.3	67; 25.7 ± 0.5^@^	33; 22.2 ± 1.1^@^	13; 20.8 ± 1.6	6; 25.6 ± 3.5	4; 30.5 ± 0.2
S1-CGRP stain	392; 19.3 ± 0.2	106; 23.4 ± 0.7	183; 19.6 ± 0.4	73; 23.6 ± 0.6	13; 23.0 ± 1.8	26; 23.9 ± 1.5
Total LS	1592; 18.5 + 0.2##	315; 22.7 + 0.4	770; 18.6 + 0.2##	316; 21.5 + 0.4	77; 19.8 ± 0.7^##^	76; 23.0 + 0.9

Data displayed as “n; mean cell diameter in μm ± SEM”. bPANS indicates bladder Primary Afferent Neurons.

FB indicates labeling following intraparenchymal injection of Fast Blue dye. DiI indicates labeling following intravesical injection of DiI. Peptide indicates labelling using anti-bodies to the indicated peptide. DRG indicates Dorsal Root Ganglion.

IHC indicates specific immunohistochemical technique applied.

CGRP indicates calcitonin gene-related peptide and SP indicates Substance P.

*&** indicate statistical difference from corresponding Total LS measure with p < 0.05 & p < 0.01 respectively.

^#^&^##^ indicate statistical difference from corresponding peptide measure with p < 0.05 & p < 0.01 respectively.

^@^&^@@^ indicate statistical difference from corresponding CGRP group with p < 0.05 & p < 0.01 respectively.

### 3.2 Characteristics of thoracolumbar (TL) bladder afferent neurons

The number of bPANs retrogradely labeled by intraparenchymal and intravesical dye administration was quantified in histological T13 and L1 DRG sections. The route of dye administration had a profound effect on the number of labeled neurons with 915 neurons (85.6% of TL sample) identified as having exclusively Non-Urothelial nerve endings and only 121 neurons (11.3% of TL sample) identified as having exclusively Urothelial nerve endings. The sample of bPANs with both Urothelial and.

Non-Urothelial labeling was extremely small, with only 33 neurons double labeled (3.1% of labeled TL neurons). Cell diameters were compared for the individually labeled neuronal groups and demonstrated that the mean diameter of TL Urothelial only bPANs was 21.8 ± 0.7 μm while the mean diameter of TL Non-Urothelial only bPANs was significantly larger at 23.8 ± 0.2 μm (P = 0.0033). Notably, the cell diameters of the double-labeled bPANs was even larger at 26.6 ± 1.0 μm, which was statistically larger than either of the other samples (comparison with Non-Urothelial only sample p = 0.0148; with Urothelial only sample p < 0.0001).

Peptidergic labeling also differed between TL Urothelial and Non-Urothelial bPANs. Whereas only 6.6% of TL Urothelial bPANs were identified as expressing SP or CGRP, 14.8% of TL Non-Urothelial bPANs were identified as expressing SP or CGRP. This difference in expression was statistically significant (p = 0.0116). The double-labeled bPANs had the highest number expressing SP or CGRP with 75.8% of those neurons identified as peptidergic (p < 0.0001 versus Urothelial bPANs; p < 0.0001 versus Non-Urothelial bPANs). Cell diameters of peptidergic versus nonpeptidergic neurons (as listed in Table 1 and displayed graphically in Fig. 3) did not significantly differ in the TL Non-Urothelial bPANs or in the double-labeled bPANs, but there was a trend in the limited sample (n = 8) of peptidergic TL Urothelial bPANs to be larger (p = 0.0578).

### 3.3 Characteristics of lumbosacral (LS) bladder afferent neurons

The number of bPANs retrogradely labeled by intraparenchymal and intravesical dye administration was quantified in histological L6 and S1 DRG sections. The route of dye administration had a profound effect on the number of labeled neurons with 1907 neurons (60.6% of the total LS sample) identified as having exclusively Non-Urothelial nerve endings and 1086 neurons (34.5% of the total LS sample) identified as having Urothelial only nerve endings. The overlap between Urothelial and Non-urothelial neurons was uncommon with only 153 double-labeled LS neurons (4.9% of LS sample) identified. Cell diameters were compared for the individual labeled groups and demonstrated that the mean diameter of LS Urothelial only bPANs was 19.5 ± 0.2 μm, which did not statistically differ from the 19.3 ± 0.1 μm mean diameter of LS Non-Urothelial only bPANs. However, like the TL populations, the cell diameters of the double-labeled bPANs were statistically larger at 21.5 ± 0.6 μm than either other sample (comparison with Non-Urothelial only sample p < 0.0001; with Urothelial only sample p = 0.0004).

Peptidergic labeling also differed between LS Urothelial and Non-Urothelial bPANs. Whereas 29.1% of LS Urothelial bPANs were identified as expressing SP or CGRP, only 16.5% of LS Non-Urothelial bPANs were identified as expressing SP or CGRP. This difference in expression was statistically significant (p < 0.0001). Again, like the TL populations, the double-labeled bPANs had the highest number expressing SP or CGRP with 49.7% of those neurons identified as peptidergic (comparison with Urothelial only bPANs p < 0.0001; with Non-Urothelial only bPANs p < 0.0001). Cell diameters of peptidergic bPANs (listed in Table 1 and displayed graphically in Fig. 3) were significantly larger than those of nonpeptidergic bPANs in all three LS bPAN groups (comparison with Non-Urothelial only bPANs p < 0.0001; with Urothelial only bPANs p < 0.0001; with double-labeled bPANs p = 0.0005).

### 3.4 Comparison of bPANs at TL versus LS levels

Multiple different neuronal characteristics differed between the TL sample and the LS sample of bPANS. As apparent in Fig. 2, Urothelial-Only labeling was much more likely to occur at LS levels when compared with TL levels (34.5% vs. 11.3% incidence respectively; p < 0.0001). Peptidergic labeling was similar between TL and LS levels when examining Non-Urothelial only bPANs (14.8% vs. 16.5%) but when examining Urothelial-Only bPANs, they were much more likely to be identified as expressing CGRP or SP at LS levels than at TL levels (29.1% vs. 6.6% respectively; p < 0.0001). The cell diameters of all of the LS bPANs were significantly smaller in comparison with TL bPANs in all neuronal subgroups (see Total TL data line in Table 1).

## 4 Discussion

The most important finding of this study was its characterization of a previously unrecognized subset of bPANs that are labeled by the intravesical administration of retrograde axon-tracing dye. Previous histological and electrophysiological studies identified this “Urothelial” neuronal population only at LS levels [1,2,6], but not at TL levels. The present study gives clear evidence of the existence of these neurons but also identified differences between bPANs that project to TL spinal levels when compared with bPANs that project to LS spinal levels. LS bPANs had smaller diameters and were more peptidergic in number than TL bPANs. Urothelial bPANs formed a much smaller percentage of the total sample of TL bPANs than LS bPANs and had very limited immunohistochemical labeling for SubP or CGRP at the TL levels versus at the LS levels, where peptidergic neurons formed a third of the Urothelial bPAN sample. Similar to our previous studies [1,2], there was very little double labeling of bPANs from both Urothelial and Non-Urothelial dye injection sites, a phenomenon present at both the TL and LS levels. Interestingly, the double-labeled bPANs were statistically larger in diameter than the single-labeled bPANs at both the TL and LS spinal levels. Based on a limited sampling of rat DRG neurons, a soma diameter of ≤30 μm has been used as a correlate to identify C-fiber, and some Aδ-fiber, neurons [7]; virtually all of the studied neurons in the present study had diameters <30 μm and so are likely associated with slowly conducting primary afferent nerve fibers. This is consistent with the majority of pelvic visceral organ sensory innervation being by C-fiber and Aδ-fiber type neurons, and with previously published cell size of bladder-innervating DRG neurons [8].

Others have also identified differences between TL and LS visceral afferents, such as differences in their chemosensitivity [[9], [10], [11], [12]], neurochemical expression [13,14], gene expression profiles [15], and transmission to ascending pathways [16]. Each of these differences may contribute to the unique functional responses to normal physiologic stimuli and inflammatory stimuli that are exhibited by LS and TL afferent neurons [[6], [17], [18], [19], [20]]. Previous studies have examined SP and CGRP expression in bPANs. The neuropeptide expression data reported here represent a smaller proportion of LS peptidergic neurons than that previously reported. However, the criterion for labeling was very stringent in the present study (>5× background) and so likely represents an undercounting of the total number of peptidergic neurons when compared to other studies (e.g. Refs. [21,22]).

An important next step in the characterization of these different neuronal populations is to determine, using bPAN electrophysiological measures or other techniques (e.g., calcium imaging), whether their responsiveness to various mechanical and chemical stimuli applied to the bladder or *in vitro* to their cell bodies may differ. The proportion of Urothelial LS bPANs identified and characterized in the present study may be similar to a functionally identified population in the mouse that responds to light stroking of the urothelium (i.e., mucosal afferents) but not to circumferential stretch (i.e., muscular afferents), as previously reported by Xu and Gebhart [6]. This functionally identified population comprised 9% of all characterized neurons, a proportion similar to what we have previously reported in the same species [2]. However, this population was only observed in pelvic nerve (i.e., LS) and not lumbar splanchnic (i.e., TL) bladder primary afferents, whereas TL Urothelial bPANs were identified in the present study. This discrepancy may be due to species differences, since the present study was conducted in rat and the aforementioned study was in mouse. However, so-called mucosal afferents, which are perhaps analogous to Urothelial afferents described here, have been identified in the lumbar splanchnic innervation of the distal colon [23]. Further, it is possible that the mucosal afferents identified via single fiber recordings that do not normally respond to bladder stretch comprise a group of mechanically insensitive afferents (MIAs or ‘silent’ afferents), or a subset of this group. MIAs comprise approximately 30% of lumbar splanchnic afferents innervating the distal colon [23].

The urothelium is an active component of bladder sensation and function that has been associated with lower urinary tract pathologies. Urothelial cells release neurochemicals in response to mechanical and chemical stimuli that, in turn, modulate afferent nerve activity (e.g. Refs. [[24], [25], [26], [27], [28]], and others; see Ref. [29] for review). Afferents with terminal endings in proximity to the urothelium have been shown to express ligand-gated receptors for urothelial-derived mediators known to alter afferent signaling [30]. The Urothelial bPAN population described herein likely comprise such an afferent group.

In conclusion, the present study gave additional neuroanatomical evidence of differences between Urothelial and Non-Urothelial bPANs and extended these differences to include not only LS but TL neurons. Any neurophysiological understanding of bladder sensation needs to recognize that these different populations of neurons exist, which likely subserve different physiological functions. Older neuroanatomical/neurophysiological data exist which will need to be re-examined, as the methodology they utilized would only have labeled Non-Urothelial bPANs (e.g. Ref. [5]) further demonstrating that our understanding of Urothelial bPANs is incomplete.

## Funding

United States National Institutes of Health: DK51413.

## Author contribution statement

Buffie Clodfelder-Miller: Conceived and designed the experiments; Performed the experiments; Analyzed and interpreted the data; Wrote the paper. Jennifer J. DeBerry: Conceived and designed the experiments; Analyzed and interpreted the data; Wrote the paper. Timothy J. Ness: Conceived and designed the experiments; Analyzed and interpreted the data; Contributed reagents, materials, analysis tools or data; Wrote the paper.

## Data availability statement

Data will be made available on request.

## Declaration of competing interest

The authors declare that they have no known competing financial interests or personal relationships that could have appeared to influence the work reported in this paper.
